# Effects of High-Intensity Interval Training vs Moderate-Intensity Continuous Training on Body Composition and Blood Biomarkers in Coronary Artery Disease Patients: A Randomized Controlled Trial

**DOI:** 10.31083/j.rcm2503102

**Published:** 2024-03-11

**Authors:** Catarina Gonçalves, Armando Raimundo, Ana Abreu, João Pais, Jorge Bravo

**Affiliations:** ^1^Departamento de Desporto e Saúde, Escola de Saúde e Desenvolvimento Humano, Universidade de Évora, 7000-727 Évora, Portugal; ^2^Comprehensive Health Research Centre, 7002 - 554 Évora, Portugal; ^3^Department of Cardiology, Santa Maria Hospital, 1649-028 Lisbon, Portugal; ^4^Department of Cardiology, Espírito Santo Hospital of Évora, 7000-811 Évora, Portugal

**Keywords:** cardiovascular disease, cardiovascular risk factors, clinical trials, high-intensity interval training, randomized controlled trial

## Abstract

**Background:**

Cardiac rehabilitation (CR) is essential in reducing cardiovascular mortality and morbidity. High-intensity interval training (HIIT) has emerged as a promising exercise intervention for enhancing clinical outcomes in cardiac patients. This study aimed to investigate the effects of two short-term exercise-based programs employing HIIT and moderate-intensity continuous training (MICT) in comparison to a control group concerning blood pressure, body composition, and blood biomarkers in patients diagnosed with coronary artery disease (CAD).

**Methods:**

Seventy-two CAD patients (14% women) underwent randomization into three groups: HIIT, MICT, and control. The training programs encompassed six weeks of supervised treadmill exercises, conducted thrice weekly. MICT targeted ≈70–75% of peak heart rate (HRpeak), while HIIT was tailored to ≈85–95% of HRpeak. The control group received guidance on adopting healthy lifestyles. Outcome measurements included evaluations of blood pressure, body composition, and blood biomarkers.

**Results:**

In contrast to MICT, the HIIT exhibited superior improvements in body fat mass (Δ%HIIT: 4.5%, *p* < 0.001 vs. Δ%MICT: 3.2%, *p* < 0.001), waist circumference (Δ%HIIT: 4.1%, *p* = 0.002 vs. Δ%MICT: 2.5%, *p* = 0.002), hemoglobin A1c (HbA1c) (Δ%HIIT: 10.4%, *p* < 0.001 vs. Δ%MICT: 32.3%, *p* < 0.001) and thyrotropin (TSH) (Δ%HIIT: 16.5%, *p* = 0.007 vs. Δ%MICT: 3.1%, *p* = 0.201). Both HIIT and MICT induced significant enhancements across all variables compared to the control group.

**Conclusions:**

HIIT and MICT emerged as effective modalities for enhancing systolic and diastolic function, body composition, and blood biomarkers in CAD patients, with HIIT demonstrating incremental improvements over MICT. The absence of participation in exercise-based programs following cardiovascular events yielded less favorable outcomes. HIIT holds promise as an adjunct intervention in CR programs for CAD patients.

**Clinical Trial Registration:**

https://clinicaltrials.gov/ct2/show/NCT03538119.

## 1. Introduction

Cardiovascular disease (CVD) stands as the predominant global cause of mortality, contributing to a substantial 30% of all recorded deaths (16.7 million individuals) [1]. Within the ambit of CVD, coronary artery disease (CAD) emerges as the most prevalent etiology in CVD-related fatalities. Forecasts indicate a looming surge of 16.6% in CAD-related mortalities by the year 2030 [2]. Consequently, the implementation of effective strategies to mitigate the impact of CVD assumes paramount importance. Among these strategies, comprehensive exercise-based cardiac rehabilitation (CR) has garnered worldwide acceptance as a potent secondary prevention tool for patients with various forms of CVD. A key component of a CR program is exercise training which has demonstrated its efficacy in not only reducing mortality rates but also augmenting the quality of life, ameliorating frailty, and enhancing cardiovascular fitness (defined as peak oxygen uptake [${\mathrm{VO}}_{2}$]), a parameter recognized as an autonomous predictor of hospitalizations and mortality in patients afflicted with CVD [3].

Comprehensive CR programs encompass distinct phases designed to facilitate patients’ transition from acute hospital care (Phase I) to the resumption of their daily activities, spanning phases II (subacute), III (outpatient), and IV (maintenance). The World Health Organization (WHO) recognizes the multifaceted impact of exercise-based CR on patients, acknowledging its potential to influence their physical, psychological, and social well-being, enhance their overall quality of life, and mitigate the risk of potential complications [1]. Moreover, the implementation of safe exercise protocols, tailored to various intensity levels, exerts discernible effects on training endurance, oxygen capacity, and intervention outcomes. Notably, extant research has evidenced the favorable impact of exercise-based CR on a spectrum of physiological and clinical parameters, including blood pressure [2, 4], blood lipids [2, 4], insulin dynamics [2, 4], physical fitness [5, 6], body composition [7, 8, 9], heart rate variability (HRV) [10, 11, 12] and health-related quality of life [13, 14].

Moderate-intensity continuous training (MICT) has historically served as a cornerstone in the prescription of aerobic-based exercise, typically consisting of 30–60 min, targeting an intensity range of 50–75% of heart rate (HR) [15]. This approach has demonstrated both short-term and enduring clinical benefits for individuals afflicted with CVD [16]. Notwithstanding these advantages, a noteworthy proportion of the adult population, approximately 30%, grapples with an inability to fulfill this exercise regimen due to constraints such as time scarcity [17]. The protracted duration and intricate nature of MICT can contribute to patient attrition, rendering exercise compliance challenging [18]. Conversely, high-intensity interval training (HIIT) has recently emerged as an alternative or supplementary strategy to MICT. HIIT entails recurring bouts of relatively elevated exercise intensity, typically within the range of 85–100%, interspersed with intervals of lower-intensity recovery, totaling 20–30 min of exercise [19]. Notably, HIIT has exhibited the capacity to yield comparable or even superior enhancements in ${\mathrm{VO}}_{2}$ in comparison to MICT [16, 17, 18, 19, 20]. Indeed, HIIT has demonstrated effectiveness on par with, if not surpassing, MICT in terms of its capacity to ameliorate clinical outcomes in CVD patients, encompassing improvements in body composition [21], HR response to exercise [22], and myocardial function [23]. Crucially, HIIT also appears to be as safe as MICT among older individuals undergoing CR [24, 25].

Despite the pronounced health enhancements associated with CR, it is disconcerting that less than 8% of survivors of various CVD are enrolled in CR programs within Portugal, and among those who do enrol, adherence rates remain notably suboptimal [26]. Regrettably, the dearth of exercise-based CR initiatives in the country exacerbates this situation, with a glaring paucity in the geographical dispersion of these facilities. Notably, the absence of any CR center in the Alentejo region, where the prevalence of CVD is notably elevated, accentuates this concern. Moreover, while the merits of HIIT have gradually emerged, there exists a notable dearth of research elucidating the role and validity of HIIT in the context of CAD patients within the country. Hence, the primary objective of the present study is to scrutinize the ramifications of two distinct six-week exercise-based regimens, namely HIIT and MICT, with regard to their impacts on body composition and cardiovascular biomarkers, while concurrently assessing risk factors. These outcomes will be juxtaposed against those of a control group.

## 2. Methods

This study is a single-blinded randomized controlled trial (RCT) and followed the Consolidated Standards of Reporting Trials (CONSORT) guidelines for RCTs (http://www.consort-statement.org).

### 2.1 Participants

Three hundred and eight patients were enrolled in the study between March 2018 and November 2021, at the cardiology unit of the Espírito Santo Hospital of Évora, Portugal. The study included patients who had suffered a coronary event and were referred to the community-based exercise programs by their cardiologist, two months after angioplasty. Patients between the ages of 18 and 80, with a left ventricular ejection fraction ≥45%, and classified as New York Heart Association (NYHA) functional Class I or II were considered for inclusion. Patients who had severe exercise intolerance, uncontrolled angina pectoris, uncontrolled arrhythmia, lung or severe kidney diseases, musculoskeletal or neuromuscular conditions preventing exercise testing and training, and signs or symptoms of ischemia were excluded from the study. Recruitment ended once the required sample size for the primary outcome was reached. All patients completed a medical history and health questionnaire and provided written informed consent.

#### Randomization and Masking 

**Fig. 1.**
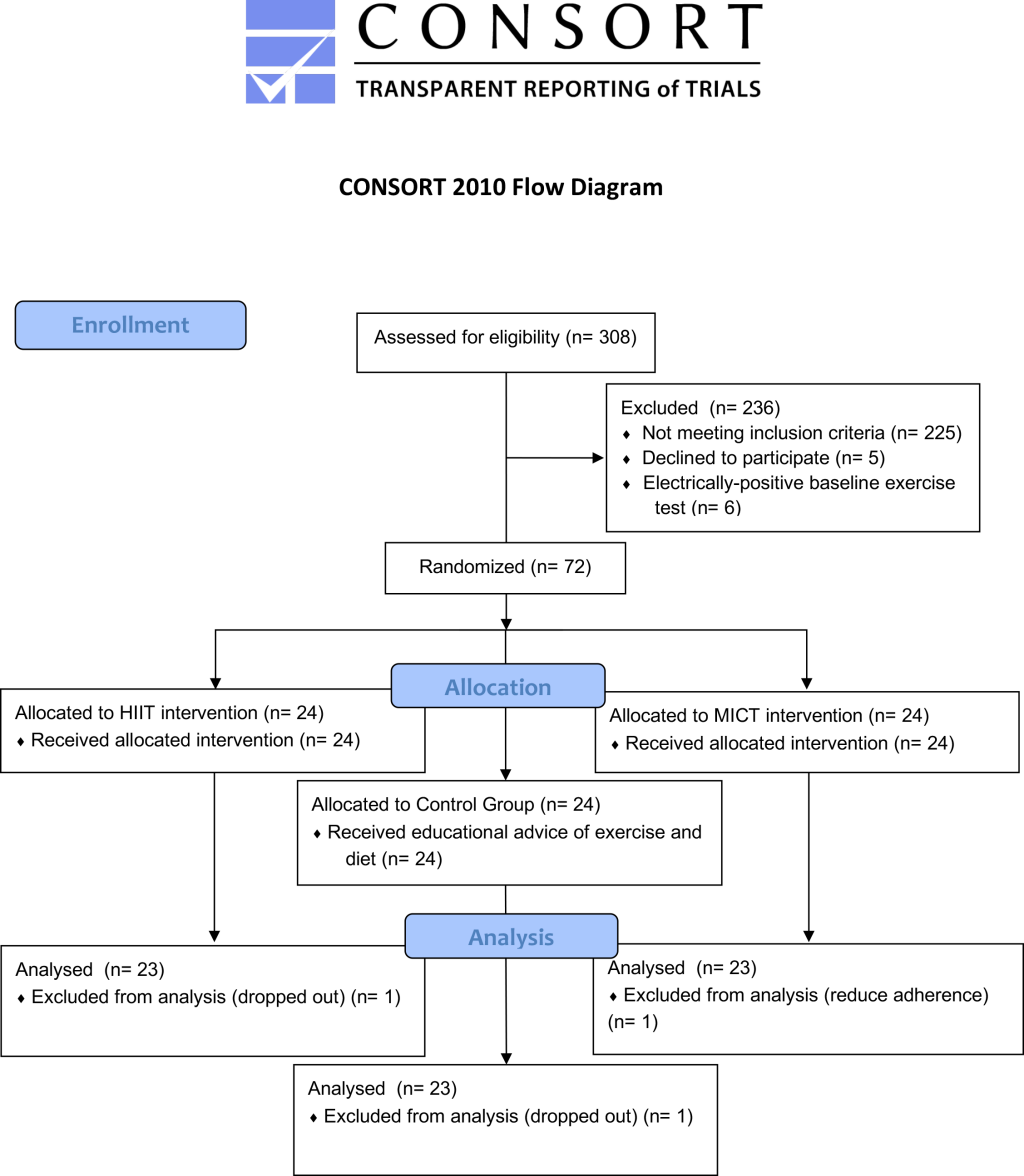
**Diagram of the study**. HIIT, high-intensity interval training; MICT, Moderate-intensity continuous training.

After the baseline assessment and before the start of community-based exercise programs, the 72 patients were randomly assigned in a 1:1:1 allocation ratio to one of three groups: HIIT, MICT (traditional), and control (usual medical recommendations) (Fig. 1). To ensure that allocation concealment was maintained, patients belonging to each group were scheduled to be seen at specific, separate times that did not coincide with appointments for patients in the other groups. The three groups were carefully matched in terms of age, extent of coronary artery disease, coronary risk factors, type of coronary event, and left ventricular ejection fraction. While patients and physicians assigned to the intervention group were aware of their allocated category, outcome assessors and data analysts remained blinded to the allocation throughout the study.

### 2.2 Outcome Measures and Assessments

#### 2.2.1 Exercise Testing 

Initially, the CAD patients were submitted to a clinical evaluation performed by a cardiologist. A supervised graded exercise test to record volitional fatigue, risks or symptoms of ischemia was performed on a treadmill with the Bruce protocol [27] before the six-week intervention period. The test was done in non-fasting conditions and under medication. Electrocardiography was recorded continuously, and blood pressure was measured with an arm cuff every three minutes. Functional capacity in metabolic equivalents (METs) value was calculated. As a high proportion of patients with CAD are prescribed beta-blocker therapy, this relative method of exercise intensity takes into account the likely lower peak heart rate (HRpeak) achieved by these patients during the exercise test. To ensure training exercise intensity was reflective of medication effects, all patients were instructed to take their usual medications before the maximal exercise test.

Exercise capacity was considered as peak oxygen consumed (${\mathrm{VO}}_{2}$peak, mL/kg/min) that was directly measured by performing a cardiopulmonary exercise test. ${\mathrm{VO}}_{2}$peak was calculated using the formula: *${\mathrm{VO}}_{2}$peak = 3.5 mL/kg/min × peak METs * [28] which was determined by the standard exercise stress test (HIIT = 23; MICT = 23; Control = 23).

#### 2.2.2 Biomarkers 

Blood samples were collected on the same day as the exercise testing, but before the exercise. The final blood samples were collected 24–48 hours after the last exercise session. Levels of various biomarkers such as total cholesterol, low-density lipoprotein cholesterol (LDL-C), high-density lipoprotein cholesterol (HDL-C), triglycerides (TG), high-sensitive C-reactive protein (hsCRP), fasting blood glucose (FBG), hemoglobin A1c (HbA1c), higher free thyroxine (T4), and lower total triiodothyronine (T3), were measured. Blood samples were drawn at the beginning and at the end of the study.

#### 2.2.3 Body Composition and Risk Factor Screening 

On the second visit, the patients were submitted to a clinical evaluation of body composition performed by a physiologist at the laboratory of the University of Evora. Patients were asked to bring any medications that they were taking to the assessments. Initially, each patient completed a standardized questionnaire including medical history, medication use, demographic data, smoking status, and family history of CVD. Body mass index (BMI) was calculated directly by the standard formula: *weight (kg) / height ${\mathrm{(m)}}^{2}$*, and waist circumference (WC) was manually measured according to standard procedures of American College of Sports Medicine (ACSM) guidelines by a trained examiner [28, 29]. Body composition was evaluated using dual-energy x-ray absorptiometry (DXA) scans, performed with QDR 2000 densitometers (Hologic QDR, Hologic, Inc., Bedford, MA, USA) in array beam mode. The scans took place one week prior to and following the completion of 18 exercise sessions. These scans were used to determine total body mass, body fat mass, body lean mass, body fat percentage, and abdominal region fat percentage (defined as the area between the ribs and the pelvis by GE Healthcare systems) [28, 29]. Daily calibration of the scanner was completed using a manufacturer-supplied calibration block to ensure accuracy and control for potential baseline drift.

All measurements were taken at baseline and after the 6-week exercise-based programs.

### 2.3 Exercise Training Protocols

**Fig. 2.**
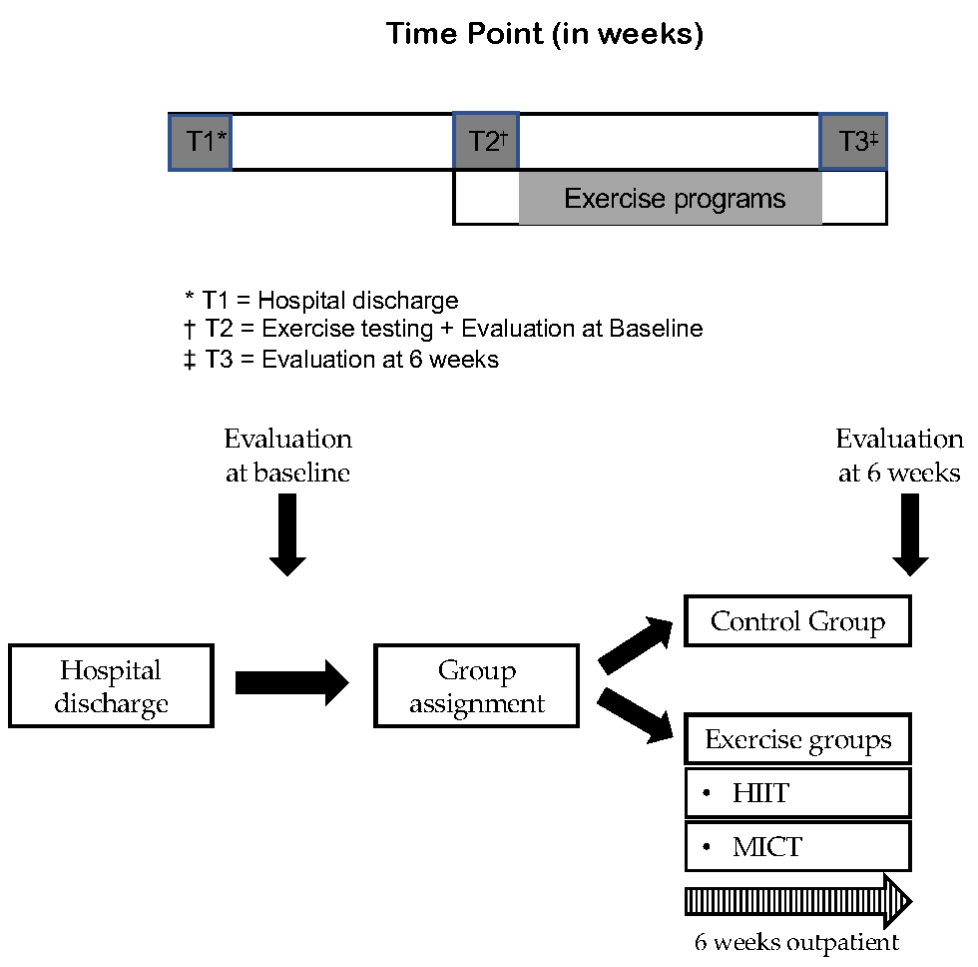
**Study design and time frame**. HIIT, high-intensity interval training; MICT, moderate-intensity continuous training; T, time point.

After hospital discharge, educational intervention, dietary advice, and psychological support were performed for all patients. The exercise programs consisted of six weeks of supervised treadmill exercise, three sessions per week (Fig. 2). If a session was missed, it was made up that week or the following week. Patients performed each exercise session in a group, including a maximum of three patients per session.

The exercise intensity was calculated using the following heart rate reserve (HRR) equation: *Target HR = [(HRmax (Maximum Heart Rate) – HRrest (Resting Heart Rate))] × %intensity desired + HRrest * [28], predicted with a supervised graded exercise test on a treadmill (Bruce protocol) [27]. Training sessions were supervised by a physiologist. Blood pressure was measured at the beginning and end of each session. The patients’ heart rate, rate of perceived exertion (measured using the Borg Scale) [30], and cardiac symptoms were all taken into account as training intensity increased. Heart rates were monitored using Polar heart rate monitoring equipment (Polar Electro Oy, Kempele, Finland). During the exercise, patients were asked to rate their perceived effort using the 10-point Category-Ratio Borg Scale [30], commonly known as the Rating of Perceived Exertion (RPE). This scale ranges from 0 to 10 with anchors ranging from ‘No exertion at all’ (0) to ‘Maximal exertion’ (10). Patients were required to rate their exertion before the exercise, immediately after each minute, and at the end of the exercise. Buchheit *et al*. [31] and Levinger *et al*. [32] have shown that the Borg Scale has a strong correlation with HR, ventilation, and ${\mathrm{VO}}_{2}$peak in individuals with CAD. The correlation is not impacted by beta-blocker medication, which is commonly used by patients with CAD to modulate their HR [29]. During exercise, patients’ heart rate was monitored minute-to-minute using a H10 chest strap manufactured by Polar Inc. (Kempele, Finland).

**Fig. 3.**
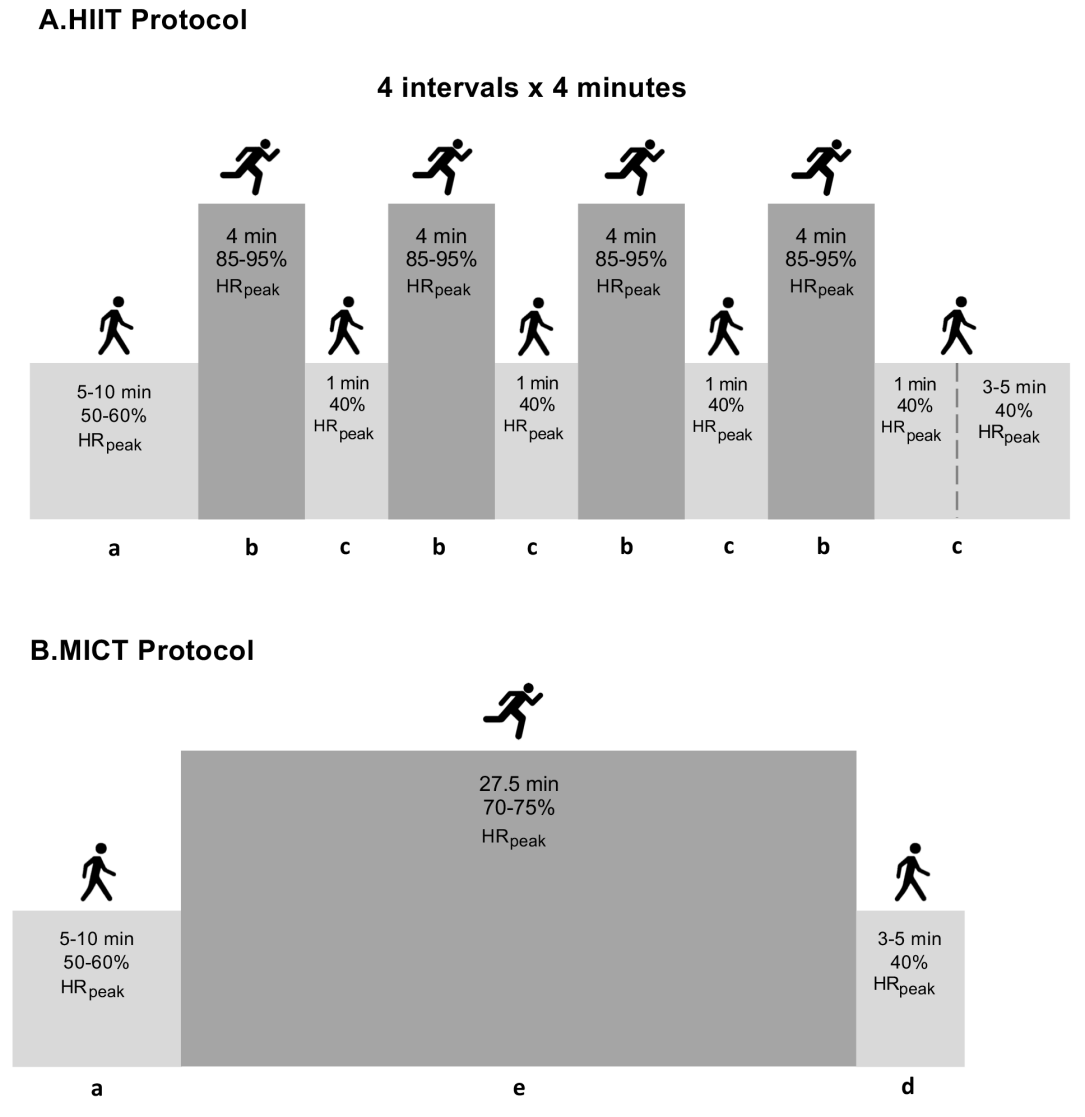
**Summary of the exercise training protocols**. Detailed description of exercise training protocol elsewhere [10]. Abbreviations: a, warm-up; b, interval bout of high-intensity exercise; c, one-minute recovery interval; d, cool-down; e, continuous bout of moderate-intensity exercise; HIIT, high-intensity interval training; MICT, moderate-continuous training; min, minutes; HRpeak, peak heart rate.

Explanations for part labels A and B are ungiven in the Fig. 3 caption. Each exercise session was initiated with a 5–10-minute warm-up at 50–60% HRpeak and finished with 5 minutes of cool-down at 40% HRpeak. The HIIT group performed 4 × 4-minute high-intensity intervals at 85%–95% HRpeak followed by a 1-minute recovery interval at 40% HRpeak, predicted with the Bruce protocol [27]. Throughout the exercise, the patients were motivated to gradually increase their exercise intensity towards 6–9 (hard to very hard) on a 0 to 10 Borg scale. The MICT group (traditional care) performed a continuous bout of moderate-intensity exercise at 70–75% HRpeak, rating perceived exertion 3 to 5 (fairly light to somewhat hard), for 28 minutes in order to equate the energy expenditure with the HIIT group (Fig. 3, Ref. [10]). The information about the mean of patients’ heart rate and rate of perceived exertion (Borg scale) pre-post session throughout the six weeks of both exercise-based programs can be seen in the **Supplementary Table 1**. The control group did not receive any additional follow-up regarding exercise beyond general advice on the importance of exercise and diet.

### 2.4 Statistical Analyses

The sample size was calculated using the online G*Power software (University of Dusseldorf, Dusseldorf, Germany), considering an effect size of 0.3, a predefined sample power of 0.8, a predefined error probability defined as 0.05, and a statistical power of 95% [33]. As a result, we determined that a minimum sample size of 66 participants (22 participants for each group) was necessary to identify significant changes.

The normality and homogeneity assumptions were tested using the Kolmogorov-Smirnov and Levene tests, respectively. Since the majority of sample variables did not conform to a normal distribution, non-parametric statistical analyses were used. Between-group comparisons were performed using the Kruskal-Wallis test, while within-group comparisons were performed using the Friedman test. Both tests were then followed by post hoc pairwise comparisons.

The means and standard deviations were calculated for all variables. The delta value *(Δ: ${\mathrm{moment}}_{x}$ – ${\mathrm{momentx}}_{-1}$)* and the proportional change delta value *(Δ%: [(${\mathrm{moment}}_{x}$ – ${\mathrm{momentx}}_{-1}$) / ${\mathrm{momentx}}_{-1}$] × 100)* were calculated for all variables to compare post-intervention values with baseline values.

The effect size (ES) was calculated using Cohen’s method since the data did not follow a normal distribution [33]. The ES was classified based on Cohen’s thresholds (defined as small: 0.10; medium: 0.30; and large: 0.50) [34]. The analyses were performed using SPSS (version 26.0, SPSS Inc., Chicago, IL, USA). A value of *p* ≤ 0.05 was considered statistically significant for all analyses. To protect patients’ anonymity, a code was assigned to each patient.

According to the standards for dyslipidemia, we considered a HDL-C level below 50 mg/dL (for women) or below 40 mg/dL (for men), as well as a TG level of 150 mg/dL or higher, as criteria for diagnosis [35]. A hsCRP test result of 1.0 and 10.0 milligrams per deciliter (mg/dL) is defined as moderately elevated [36]. For the diagnosis of diabetes mellitus, we utilized the American Diabetic Association criteria [37]. Namely, the pre-diabetic stage was identified by HbA1c levels between 5.7 and 6.4, or impaired fasting blood glucose levels between 100 and 125 mg/dL, and diabetes mellitus was diagnosed with HbA1c ≥6.5 or fasting glucose levels ≥126 mg/dL. Impaired non-fasting glucose was defined as a glucose value of 100 mg/dL or higher [37]. Overweight was characterized by a BMI between 25.0 and 29.9 kg/$m^{2}$, while obesity was defined by a BMI of 30 kg/$m^{2}$ or higher [23]. Finally, increased WC was defined as >80 cm in women and >94 cm in men [38].

## 3. Results

The baseline characteristics of participants, as presented in Table 1, exhibited no statistically significant differences among the HIIT, MICT, and control groups: age (50 ± 9 vs. 55 ± 10 vs. 57 ± 11 years respectively, *p* = 0.180), female (15% vs. 17% vs. 15%, *p* = 0.211), and ${\mathrm{VO}}_{2}$peak (24.7 ± 9.0 vs. 23.4 ± 6.3 vs. ± 23.5 ± 11.0 mL/kg/min *p* = 0.290). Additionally, there were no significant differences in the prevalence of comorbidities or medication usage across the groups (*p* > 0.05).

**Table 1.** **Baseline characteristics of study participants**.

			Exercise-based program		No exercise-based program
			HIIT (n = 23)	MICT (n = 23)	Control (n = 23)
Demographics					
	Age (years), mean ± SD		50 ± 9	55 ± 10	57 ± 11
		>70 years, n (%)	2 (8.7)	3 (13.0)	4 (17.4)
	Gender (Male/Female)		20/3	19/4	20/3
	Retired, n (%)		2 (8.7)	7 (30.4)	7 (30.4)
	Anterior MI, n (%)		3 (13.0)	4 (17.4)	2 (8.7)
	Coronary event/intervention				
	CABG, n (%)		1 (4.3)	1 (4.3)	1 (4.3)
	PCI, n (%)		22 (95.7)	22 (95.7)	22 (95.7)
	${\mathrm{VO}}_{2}$peak (mL/kg/min), mean ± SD		24.7 ± 9.0	23.4 ± 6.3	23.5 ± 11.0
Risk factors or comorbidities					
	Diabetes mellitus, n (%)		10 (43.5)	9 (39.1)	10 (43.5)
	Hypertension, n (%)		13 (56.5)	13 (56.5)	14 (60.9)
	Dyslipidemia, n (%)		14 (60.9)	15 (65.2)	15 (65.2)
	Body Mass index (kg/$m^{2}$), mean ± SD		28.2 ± 4.5	29.4 ± 3.9	29.4 ± 4.3
	Waist Circumference (cm), mean ± SD		98.4 ± 14.5	101.1 ± 10.3	101.1 ± 10.8
	Active smoker, n (%)		6 (26.1)	4 (17.4)	4 (17.4)
	Non-smoker, but has been, n (%)		9 (39.1)	13 (56.5)	12 (52.2)
	Family history of CVD, n (%)		14 (60.9)	16 (69.6)	16 (69.6)
	Sedentarism, n (%)		13 (56.5)	19 (82.6)	19 (82.6)
	Sleep <5 h, n (%)		6 (26.1)	9 (39.1)	11 (47.8)
Current medication					
	ACE inhibitor, n (%)		21 (91.3)	23 (100)	22 (95.7)
	ARBs, n (%)		16 (69.6)	7 (73.9)	11 (47.8)
	Antiplatelet, n (%)		22 (95.7)	22 (95.7)	23 (100)
	CCBs, n (%)		2 (8.7)	5 (21.7)	5 (21.7)
	Beta-blockers, n (%)		21 (91.3)	22 (95.7)	22 (95.7)
	Diuretics, n (%)		2 (8.7)	4 (17.4)	6 (26.1)
	Insulin, n (%)		5 (21.7)	5 (21.7)	11 (47.8)
	Statin, n (%)		22 (95.7)	22 (95.7)	23 (100)

CABG, coronary artery bypass graft; PCI, percutaneous coronary intervention; CVD, cardiovascular disease; ACE, angiotensin-converting enzyme inhibitor; ARBs, angiotensin II receptor blockers; CCBs, calcium channel blockers; HIIT, high-intensity interval training; MI, myocardial infarction; MICT, moderate-intensity continuous training; ${\mathrm{VO}}_{2}$peak, peak oxygen consumed (measured by the cardiopulmonary exercise test). Data are reported as Mean ± Standard deviation or number and percent population (%). Significance is <0.05.

### 3.1 Resting Heart Rate and Blood Pressures

At baseline, there were no differences across groups at rest for resting HR, systolic blood pressure (SBP) or diastolic blood pressure (DBP). After six weeks, the exercise-based groups reported a significant decrease in SBP and DBP compared with the control (Table 2). The HIIT group reported a significant decrease in SBP (Δ HIIT: 9 mm Hg, *p* < 0.001) and DBP (Δ HIIT: 6 mm Hg, *p* < 0.001), and the MICT group reported similar results in SBP (Δ MICT: 8 mm Hg, *p* < 0.001) and equal results in DBP (Δ MICT: 6 mm Hg, *p* < 0.001). The corresponding ES in resting HR was medium between the baseline and the post-intervention periods in both exercise groups (HIIT *d *= 0.56 and MICT *d *= 0.55), and in SBP and DBP were large in both exercise groups (HIIT *d* = 1.27 and MICT *d* = 0.86; HIIT *d* = 1.11 and MICT *d* = 1.19, respectively).

**Table 2.** **Blood profile measurements of exercise groups and control group**.

		Baseline (A)	6-week (B)	*p*-value	ES (95% CI)	Pairwise comparison
Resting HR (bpm)						
	HIIT (n = 23)	70 ± 15.4	63 ± 8.7	0.061	–0.556 (–0.918; –0.194)	-
	MICT (n = 23)	67 ± 9.4	62 ± 4.9	0.020	–0.551 (–1.060; –0.043)	-
	Control (n = 23)	69 ± 9.9	68 ± 8.1	0.835	–0.202 (–0.459; 0.054)	-
SBP (mm Hg)						
	HIIT (n = 23)	135 ± 12.1	121 ± 9.5	<0.001 ^a^	–1.270 (–1.825; –0.715)	A > B
	MICT (n = 23)	135 ± 13.3	125 ± 10.9	<0.001 ^b^	–0.861 (–1.208; –0.514)	A > B
	Control (n = 23)	139 ± 6.1	136 ± 8.2	0.297	–0.439 (–0.914; 0.035)	-
DBP (mm Hg)						
	HIIT (n = 23)	95 ± 11.6	88 ± 8.4	<0.001 ^a^	–1.106 (–1.624; –0.587)	A > B
	MICT (n = 23)	94 ± 9.6	89 ± 7.4	<0.001 ^b^	–1.191 (–1.659; –0.722)	A > B
	Control (n = 23)	95 ± 6.3	97 ± 4.9	0.144	0.360 (0.092; 1.052)	-

bpm, beats per minute; DBP, diastolic blood pressure; ES, effect size; HIIT, high-intensity interval training; HR, heart rate; MICT, moderate-intensity continuous training; SBP, systolic blood pressure. Values are reported as Mean ± Standard deviation. ^a^ significant differences between HIIT and Control, *p* < 0.05; ^b^ significant differences between MICT and Control, *p* < 0.05. </>/= indicates whether HIIT, MICT or Control achieved a more desirable outcome.

### 3.2 Body Composition Measurements 

At baseline, there were no differences across groups in body composition measurements. Following six weeks of exercise, the results (Table 3) showed that the HIIT group demonstrated significant improvements compared to MICT in body fat mass (Δ% HIIT: 4.5%, *p* < 0.001 vs. Δ% MICT: 3.2%, *p* < 0.001), and waist circumference (Δ% HIIT: 4.1%, *p* = 0.002 vs. Δ% MICT: 2.5%, *p* = 0.002). The control group had no improvements. On the other hand, all values of body composition measurements increased from baseline to post-intervention. The respective ES from baseline to six weeks were small in the HIIT group in body weight (*d* = 0.20), abdominal fat percentage (*d* = 0.28) and BMI (*d *= 0.22), and medium in waist circumference (*d *= 0.34). Moreover, in the MICT group, the effect sizes were small in body fat percentage (*d* = 0.22), total body fat mass (*d* = 0.22) and waist circumference (*d *= 0.22).

**Table 3.** **Body composition measurements of exercise groups and control group**.

		Baseline (A)	6-week (B)	*p*-value	ES (95% CI)	Pairwise comparison
Body weight (kg)						
	HIIT (n = 23)	82.6 ± 14.5	79.9 ± 12.8	<0.001	–0.202 (–0.331; –0.073)	-
	MICT (n = 23)	81.9 ± 11.7	81.1 ± 11.2	0.003	–0.072 (–0.160; 0.016)	-
	Control (n = 23)	83.1 ± 13.9	83.6 ± 14.7	0.513	0.010 (–0.060; 0.079)	-
BMI (kg/$m^{2}$)						
	HIIT (n = 23)	28.2 ± 4.5	27.2 ± 3.8	<0.001	–0.221 (–0.358; –0.085)	-
	MICT (n = 23)	29.5 ± 3.9	29.2 ± 3.9	0.005	–0.062 (–0.150; 0.026)	-
	Control (n = 23)	29.4 ± 4.3	29.5 ± 4.4	0.655	0.014 (–0.074; 0.102)	-
Body fat (%)						
	HIIT (n = 23)	28.2 ± 5.3	27.0 ± 5.5	0.002 ^a^	–0.186 (–0.280; –0.092)	A > B
	MICT (n = 23)	32.6 ± 6.0	31.2 ± 5.6	<0.001 ^b^	–0.215 (–0.340; –0.089)	A > B
	Control (n = 23)	29.7 ± 5.0	30.0 ± 4.8	0.827	0.025 (–0.063; 0.114)	-
Body fat mass (kg)						
	HIIT (n = 23)	23.1 ± 67.6	22.0 ± 67.3	<0.001 ^a,c^	–0.146 (–0.236; –0.026)	A > B
	MICT (n = 23)	25.7 ± 48.7	24.7 ± 42.1	<0.001 ^b^	–0.217 (–0.377; –0.057)	A > B
	Control (n = 23)	24.8 ± 60.9	25.3 ± 56.0	0.061	0.089 (0.021; 0.158)	-
Abdominal fat (%)						
	HIIT (n = 23)	36.3 ± 6.9	34.5 ± 5.9	<0.001 ^a^	–0.283 (–0.427; –0.138)	A > B
	MICT (n = 23)	37.4 ± 7.1	36.1 ± 6.4	<0.001 ^b^	–0.192 (–0.285; –0.099)	A > B
	Control (n = 23)	37.4 ± 6.0	38.4 ± 6.8	0.023	0.165 (0.059; 0.271)	-
Lean mass (kg)						
	HIIT (n = 23)	54.7 ± 14.6	55.3 ± 15.0	0.144	0.041 (–0.034; 0.117)	-
	MICT (n = 23)	55.7 ± 9.7	56.4 ± 10.0	0.007	0.130 (0.025; 0.235)	-
	Control (n = 23)	56.6 ± 12.3	56.9 ± 12.9	0.835	0.021 (–0.031; 0.072)	-
WC (cm)						
	HIIT (n = 23)	98.3 ± 14.4	93.8 ± 11.4	0.002 ^a,c^	–0.341 (–0.563; –0.119)	A > B
	MICT (n = 23)	101.0 ± 10.6	98.3 ± 9.0	0.002 ^b^	–0.272 (–0.456; –0.088)	A > B
	Control (n = 23)	101.7 ± 10.4	102.8 ± 10.5	0.491	0.002 (–0.139; 0.144)	-

BMI, body mass index; ES, effect size; HIIT, high-intensity interval training; MICT, moderate-intensity continuous training; WC, waist circumference. Values are reported as Mean ± Standard deviation. ^a^ significant differences between HIIT and Control, *p* < 0.05; ^b^ significant differences between MICT and Control, *p* < 0.05; ^c^ significant differences between HIIT and MICT, *p* < 0.05. </>/= indicates whether HIIT, MICT or Control achieved a more desirable outcome.

### 3.3 Blood Biomarkers

Concerning blood biomarkers (Table 4), there were no differences across groups at baseline, but significant within-group changes between the baseline and the post-intervention were observed in both exercise protocols. The HIIT group revealed significant results comparing to MICT in HbA1c (Δ% HIIT: 10.4%, *p* < 0.001 vs. Δ% MICT: 32.3%, *p* < 0.001) and thyrotropin (TSH) (Δ% HIIT: 16.5%, *p* = 0.007 vs. Δ% MICT: 3.1%, *p* = 0.201). After the 6-week intervention, the control group had worse results, except for cholesterol variables, namely, in HDL-C (Δ% control: 15.9%, *p* = 0.002). However, it continues to be considered dyslipidemia as defined by the American College of Cardiology, although the exercise-based groups improved the lipid profile levels from baseline to post-intervention to very close to normal. The same was verified in the blood sugar and thyroid variables in the exercise-based groups but not in the control group.

**Table 4.** **Blood biomarkers of exercise groups and control group**.

		Baseline (A)	6-week (B)	*p*-value	ES (95% CI)	Pairwise comparison
Total cholesterol (mmol/L)						
	HIIT (n = 23)	175 ± 35.2	151 ± 21.8	<0.001 ^a^	–1.351 (–1.198; –0.714)	A > B
	MICT (n = 23)	173 ± 38.5	150 ± 30.4	<0.001	–0.677 (–1.023; –0.331)	-
	Control (n = 23)	171 ± 32.8	168 ± 38.8	0.835	–0.062 (–0.436; 0.312)	-
HDL-C (mmol/L)						
	HIIT (n = 23)	43 ± 6.7	54 ± 12.3	<0.001 ^a^	1.170 (0.640; 1.701)	A < B
	MICT (n = 23)	43 ± 9.0	52 ± 9.4	<0.001 ^b^	1.053 (0.598; 1.508)	A < B
	Control (n = 23)	40 ± 9.1	47 ± 12.0	0.002	0.588 (0.234; 0.942)	-
LDL-C (mmol/L)						
	HIIT (n = 23)	117 ± 38.0	85 ± 32.8	<0.001 ^a^	–1.330 (–1.857; –0.804)	A > B
	MICT (n = 23)	120 ± 45.1	92 ± 39.4	<0.001 ^b^	–0.659 (–0.950; 0.367)	A > B
	Control (n = 23)	117 ± 50.4	119 ± 51.4	0.144	0.039 (–0.227; 0.304)	-
Triglycerides (mmol/L)						
	HIIT (n = 23)	200 ± 60.6	137 ± 51.2	<0.001 ^a^	–1.119 (–1.544; –0.693)	A > B
	MICT (n = 23)	187 ± 91.7	138 ± 72.1	<0.001 ^b^	–0.598 (–0.856; –0.341)	A > B
	Control (n = 23)	188 ± 78.0	187 ± 62.7	1.00	0.036 (–0.207; 0.135)	-
HbA1c (%)						
	HIIT (n = 23)	6.1± 1.3	5.4 ± 0.8	<0.001 ^a,c^	–0.645 (–0.992; –0.298)	A > B
	MICT (n = 23)	5.8 ± 0.6	5.4 ± 0.4	<0.001	–0.370 (–0.506; –0.233)	-
	Control (n = 23)	6.2 ± 0.9	6.2 ± 1.0	0.670	0.008 (–0.227; 0.227)	-
FBG (mg/dL)						
	HIIT (n = 23)	118 ± 28.3	106 ± 22.5	0.002 ^a^	–0.466 (–0.776; –0.155)	A > B
	MICT (n = 23)	114 ± 20.2	109 ± 16.2	0.007 ^b^	–0.271 (–0.537; –0.004)	A > B
	Control (n = 23)	122 ± 25.0	122 ± 29.4	0.532	0.003 (–0.245; 0.251)	-
hsCRP (mg/L)						
	HIIT (n = 23)	1.5 ± 1.7	0.4 ± 0.7	<0.001 ^a^	–0.796 (–1.312; –0.280)	A > B
	MICT (n = 23)	1.1 ±1.1	0.4 ± 0.5	<0.001 ^b^	–0.805 (–1.280; –0.329)	A > B
	Control (n = 23)	1.3 ± 0.8	1.1 ± 1.0	0.532	0.004 (–0.604; –0.004)	-
TSH (mU/L)						
	HIIT (n = 23)	1.6 ± 0.7	1.3 ± 0.9	0.007 ^a,c^	–0.407 (–0.830; 0.016)	A > B
	MICT (n = 23)	1.9 ± 0.8	1.7 ± 0.7	0.201	–0.242 (–0.543; 0.058)	-
	Control (n = 23)	1.8 ± 1.4	2.4 ± 2.2	0.007	0.089 (–0.109; 0.760)	-
T4 (ng/dL)						
	HIIT (n = 23)	0.9 ± 0.2	1.0 ± 0.1	0.006	0.439 (0.086; 0.793)	-
	MICT (n = 23)	0.9 ± 0.1	1.0 ± 0.1	0.007	0.089 (0.300; 1.138)	-
	Control (n = 23)	1.0 ± 0.4	1.1 ± 0.4	0.022	0.188 (0.035; 0.341)	-
T3 (ng/dL)						
	HIIT (n = 23)	3.7 ± 0.7	3.4 ± 0.5	0.002 ^a^	–0.465 (–0.844; –0.085)	A > B
	MICT (n = 23)	3.7 ± 0.5	3.5 ± 0.5	0.002 ^b^	–0.327 (–0.561; –0.094)	A > B
	Control (n = 23)	4.4 ± 2.6	5.3 ± 3.9	0.144	0.260 (–0.156; 0.675)	-

ES, effect size; FBG, fasting blood glucose; HDL-C, high density lipoprotein cholesterol; HIIT, high-intensity interval training; hsCRP, high-sensitive C-reactive protein; HbA1c (%), hemoglobin A1C; LDL-C, low density lipoprotein cholesterol; MICT, moderate-intensity continuous training; TSH, thyrotropin; T3, triiodothyronine; T4, thyroxin. Values are reported as Mean ± Standard deviation or number and percent population (%). ^a^ significant difference between HIIT and Control, *p* < 0.05; ^b^ significant differences between MICT and Control, *p* < 0.05; ^c^ significant differences between HIIT and MICT, *p* < 0.05. </>/= indicates whether HIIT or MICT achieved a more desirable outcome.

The respective ES from baseline to post-intervention in the HIIT group were small in FBG (*d* = 0.47) and endocrine variables: T4 (*d* = 0.44), T3 (*d* = 0.47) and TSH (*d* = 0.41); medium in HbA1c (*d* = 0.65) and hsCRP (*d* = 0.80); and large in the cholesterol variables: Total cholesterol (TC) (*d* = 1.35), HDL-C (*d* = 1.17), LDL-C (*d* = 1.33) and TG (*d* = 1.12). In the MICT group, the respective effect sizes were small in HbA1c (*d* = 0.37), FBG (*d* = 0.27), T3 (*d* = 0.33) and TSH (*d* = 0.24); medium in TC (*d* = 0.68), LDL-C (*d* = 0.66) and TG (*d* = 0.60); and large in hsCRP (*d* = 0.81) and HDL-C (*d* = 1.05).

### 3.4 Habitual Physical Activity and Diet

For habitual physical activity and dietary intake, there was no specific control. Patients just followed the ideal recommendations given by the medical specialist.

### 3.5 Adherence and Safety

Only one patient from each group discontinued the intervention, achieving 96% adherence in both groups, HIIT and MICT protocols. There were no adverse events in either protocol (HIIT and MICT) during the exercise interventions. Thus, HIIT protocols proved to be a safe, effective, and pleasant tool for low-risk patients with CAD as well.

## 4. Discussion

To our knowledge, this study represents a pioneering endeavor as an inaugural randomized controlled trial to systematically evaluate and differentiate the impacts of HIIT as opposed to MICT, in contrast to a control group, throughout a 6-week community-based exercise program in Portugal. The main findings of our study are as follows: (i) in low-risk CAD patients HIIT and MICT exercise protocols promoted a significant improvement in blood pressure profile, body weight, BMI, body fat percentage, total body fat mass, abdominal fat percentage and waist circumference, compared to the control group; (ii) blood biomarkers improvement in patients undergoing the HIIT protocol was slightly higher than MICT and mainly detected by hsCRP and TSH. In contrast, the control group had no significant improvements in these parameters. It is noteworthy that several variables exhibited an overall increase from baseline to the post-intervention phase, underscoring the systemic physiological responses engendered by exercise interventions. However, it is of significance to highlight that exceptions to this trend were observed in the form of reductions in total cholesterol and hsCRP levels from baseline to post-intervention.

Elevated blood pressure constitutes a prevalent health condition associated with heightened mortality and an augmented risk of cardiovascular disease [23]. Existing literature, as elucidated by Pattyn *et al*. [39], underscores the favorable impact of aerobic exercise on both SBP and DBP. Specifically, Cornelissen *et al*. [40] reported a reduction of 3.5 mm Hg (95% CI 2.3–4.6) and 2.5 mm Hg (95% CI 1.7–3.2) in SBP and DBP, respectively, following aerobic exercise interventions. In consonance with these findings, our study, conducted over a six-week exercise intervention period, unveiled substantial reductions in both SBP and DBP among participants in the HIIT group, with a decline of 9 mm Hg for SBP and 6 mm Hg for DBP. Similarly, the MICT group exhibited significant reductions in SBP (8 mm Hg) and DBP (6 mm Hg). Conversely, the control group demonstrated an incremental increase in SBP (1.6 mm Hg) and DBP (1 mm Hg). Remarkably, our results align with prior investigations, such as the study by Nybo *et al*. [41] which examined HIIT and MICT interventions over a 12-week period and reported notable improvements in this cardiovascular risk factor. Specifically, the HIIT group exhibited significant reductions of 8 mm Hg for SBP and 2 mm Hg for DBP, while the MICT group experienced reductions of 8 mm Hg for SBP and 5 mm Hg for DBP. Nevertheless, it is essential to acknowledge the influence of exercise intensity on blood pressure outcomes. Molmen-Hansen *et al*. [23], in their study, implemented high-intensity training at 80–90% of maximum heart rate for the HIIT group (n = 15) and moderate-intensity training at 50–70% of maximum heart rate for the MICT group (n = 19). Notably, they reported mean decreases of 12 mm Hg in SBP and 8 mm Hg in DBP for the HIIT group, whereas the MICT group achieved non-significant reductions of 4.5 mm Hg and 3.5 mm Hg in SBP and DBP, respectively. Taken together, these findings collectively underscore the potential of both aerobic exercise modalities, namely HIIT and MICT, to effectively reduce blood pressure in patients with CAD. This demonstrates their suitability for integration into the rehabilitation regimens designed for this patient population. Moreover, the observed increase in blood pressure among subjects who did not engage in any form of exercise underscores the pivotal role of exercise in the management of blood pressure in CAD patients.

Obesity, either as an independent risk factor or in conjunction with other comorbidities, significantly heightens the susceptibility to incident CAD [42]. Pertinently, measures of body fat mass and percentage have established associations with an elevated risk of cardiovascular events and all-cause mortality [43, 44]. Additionally, higher values of BMI, increased waist circumference, and augmented waist-hip ratio have been reliably linked to a heightened risk of premature mortality [2, 45, 46]. Within the framework of our RCT, we discerned a conspicuous positive influence of both HIIT and MICT on the body composition of CAD patients. Contrastingly, individuals who abstained from participating in any community-based exercise program after their cardiac event displayed tendencies towards weight gain and increased fat mass. Specifically, following a six-week intervention period within the community-based exercise program, patients in the HIIT group exhibited a weight reduction of 1.9 kg more than their counterparts in the MICT group. In contrast, the control group displayed an increment of 0.5 kg. Moreover, in terms of WC, the HIIT group demonstrated a substantial decrease of –4.5 cm, while the MICT group exhibited a decrease of –2.7 cm. In stark contrast, the control group evidenced an increase of 1.1 cm. These outcomes provide compelling evidence of the favorable impact exerted by higher-intensity exercise sessions within community-based exercise programs on body composition, which corroborates findings reported by previous studies [27, 42, 45, 46]. In our investigation, truncal fat percentage was assessed using DXA, a measure highly correlated with abdominal fat percentage [47]. Our results showcased a reduction in abdominal fat percentage, translating to a decline of 1.8% in the HIIT group and 1.3% in the MICT group, signifying a 2.8% advantage over the control group after six weeks. Previous research efforts have also probed into the efficacy of HIIT in reducing abdominal fat among CAD patients. For instance, Dun *et al*. [48] compared HIIT and MICT and reported that supervised HIIT engendered significant reductions in total fat mass, abdominal fat percentage, and an improved lipid profile in CAD patients. Similarly, Trapp *et al*. [49] conducted a comparative analysis of HIIT and MICT, finding that the HIIT group exhibited a more pronounced decrease in abdominal fat. Slightly different were the results of the study of Zhang *et al*. [50], once they demonstrated that both HIIT and MICT significantly reduced total and abdominal fat mass. It is worth noting that the study duration of six weeks in our investigation may be considered relatively short. With an extended intervention period, one could reasonably anticipate the emergence of clinically meaningful effects [48]. In the context of weight control strategies for this population, aerobic programs such as walking are crucial. The distribution of exercise intensity can affect the effectiveness of these programs. Depending on whether the load is concentrated, or continuous, different adaptations may occur due to varying levels of exertion [51]. Continuous doses can result in higher exertion for exercises with similar external intensity, while concentrating the load may lead to increased fatigue and more pronounced physiological alterations [10, 51]. For instance, a study on brisk walking in middle-aged obese females showed that both continuous and intermittent strategies were effective, but the continuous group had slightly better results in terms of weight loss and reduction of fat mass [52].

Considering the analysis of the patients’ blood biomarkers, our study results demonstrated a significant improvement in the patients of the HIIT and MICT groups. In contrast, the control group, which did not partake in any community-based exercise program, exhibited minimal changes across these biomarkers, with exceptions noted in HDL-C and T4, both of which increased. When we scrutinized patients within each exercise program, we observed strikingly similar and significant reductions in all blood lipid parameters, hsCRP, T3, and blood sugar variables for both the HIIT and MICT groups. Importantly, following the six-week intervention, the control group displayed deteriorating results across all blood variables, whereas both HIIT and MICT engendered enhancements in TC, HDL-C, LDL-C, TG, hsCRP, T3 and HbA1c. These findings bear clinical significance, particularly in the context of patients with CAD who concurrently grapple with type 2 diabetes and dyslipidemia, necessitating pharmacotherapeutic interventions. Remarkably, both exercise protocols succeeded in driving variable values back to normal levels. Our results resonate with existing literature that has juxtaposed HIIT against MICT, showcasing HIIT’s potential to induce alterations in numerous physiological and health-related markers [45]. Notably, HIIT demonstrated more pronounced improvements in total cholesterol, low-density lipoproteins, and triglycerides among CAD patients, as reported by Elmer *et al*. [53] who also observed a greater reduction in triglyceride concentrations in HIIT compared to MICT. According to Ouerghi *et al*. [54], short-term CR programs (≤10 weeks) may yield more substantial reductions in total cholesterol, LDL-C, DBP, SBP, WC, and a more substantial increase in HDL cholesterol compared to long-term CR programs. Furthermore, Pattyn *et al*. [55] provided support for the beneficial impact of aerobic exercise on variables such as WC, HDL-C, LDL-C, SBP, DBP, and BMI. Moreover, a recent meta-analysis [56] evidenced the favorable effects of lifestyle modifications on fasting blood glucose, WC, SBP and DBP, and TG, albeit with no significant impact on HDL-C.

In our study, it is noteworthy that the initial assessment revealed average levels of TSH, T3, and T4 within the normal range for all groups. Following a six-week exercise intervention, a notable trend towards further normalization of these values was observed, contrasting with a slight increase in these levels within the control group. It is crucial to underscore that subclinical hypothyroidism characterized by TSH levels exceeding 6.57 µIU/mL has been robustly linked to a significantly elevated risk of cardiovascular events and all-cause mortality [57]. Two pertinent studies showed that high TSH levels have a protective effect on stroke severity and prognosis [58, 59]. This observation underscores the importance of early intervention in cases of asymptomatic hypothyroidism, especially when TSH levels exceed or equal to 8 µIU/mL, and particularly in individuals under the age of 65 who exhibit symptoms or possess cardiac risk factors [60, 61]. Moreover, Ojamaa *et al*. [62] have demonstrated that low T3 syndrome also happens in an animal model of acute myocardial infarction (AMI), where T3 levels decreased within a week and stayed >40% lower than normal for 4 weeks, while T4 levels remained relatively stable. Similarly, Olivares *et al*. [63] reported noteworthy variations in thyroid hormone levels in post-AMI patients. Specifically, TSH levels demonstrated an increase, while T3 levels exhibited a decline lasting up to 8 weeks post-AMI. Meanwhile, T4 levels remained low for up to 12 weeks post-AMI, despite an initial surge in thyroid stimulation one week following the cardiac event. It is imperative to note that the “euthyroid reference range” for T4 typically spans from 10–28 pmol/L, while the “euthyroid range” for T3 generally falls within the interval of 4.6 to 9.7 pmol/L, with a median value of 6.63 pmol/L. Notably, a reduction in T3 levels has been associated with heightened stroke severity and increased mortality at the one-year mark [64]. Conversely, T4 levels have exhibited positive correlations with atherosclerosis in middle-aged and elderly individuals, independently of conventional cardiovascular risk factors [65]. However, it is important to highlight that only a limited number of studies have undertaken the evaluation of thyroid parameters in relation to atherosclerosis in patients with CAD. Consequently, the status of thyroid function as an independent predictor of atherosclerosis in CAD patients remains an area warranting further investigation and elucidation [58, 59, 63].

Elevated levels of HbA1c exceeding 8.5% have been established as predictive of an increased risk of all-cause CVD [65]. A normal HbA1C level is below 5.7%, whereas levels between 5.7% and 6.4% signify prediabetes, and levels at or above 6.5% indicate diabetes [65]. Notably, individuals with higher HbA1C levels within the prediabetes range are at a heightened risk of progressing to type 2 diabetes. Furthermore, it’s worth noting that hypothyroid patients often exhibit elevated HbA1c levels, which can be normalized through effective treatment addressing thyroid function, without significantly affecting FBG levels [66]. The normal range for FBG is typically 99 mg/dL or lower, while FBG levels between 100–125 mg/dL are indicative of prediabetes, and levels at or exceeding 126 mg/dL signify diabetes [66]. Within the context of our RCT, the initial assessment indicated that the average FBG and HbA1c levels of the study groups fell within the prediabetic range. However, following the six-week community-based exercise interventions, these levels exhibited a noteworthy trend towards normalization, whereas the control group experienced a marginal increase in their levels. The well-established effect of physical activity on glycemic control and body composition is corroborated by existing literature. Exercise training has been recognized as a frontline intervention for type 2 diabetes management, with numerous studies underscoring the efficacy of both HIIT [67, 68, 69] and MICT [70, 71] in effectively managing this condition. For instance, Mitranun *et al*. [72] reported that HIIT and MICT led to similar reductions in blood glucose and body fat levels among individuals with type 2 diabetes, while HbA1c levels exhibited a significant reduction with HIIT compared to MICT (*p* < 0.05). Similarly, Karstoft *et al*. [73] found that HIIT significantly reduced blood glucose and body fat levels to a greater extent (*p* < 0.05) than MICT. Consistent with our findings, HIIT emerges as slightly more effective than MICT in reducing blood glucose levels and comparable in reducing body fat. These results hold significant clinical implications, particularly given the profound repercussions of elevated blood glucose levels and obesity in the development and progression of cardiovascular diseases, such as type 2 diabetes.

High-sensitive C-reactive protein is an indicator of metabolic disorders associated with an increased risk for CVD [74]. This heightened risk is attributed to the progression of atherosclerosis, characterized by the accumulation of cholesterol on the inner linings of blood vessels and inflammation within the vessel walls [75]. Generally, a healthy hsCRP level falls below 0.9 milligrams per deciliter (mg/dL). When hsCRP test results range between 1.0 to 10.0 mg/dL, they are typically categorized as moderately elevated [36]. In our study, baseline assessments revealed that all groups exhibited moderately elevated hsCRP levels. However, following a six-week exercise intervention, both HIIT and MICT regimens succeeded in lowering hsCRP levels to within the normal range, in stark contrast to the control group, which maintained elevated values. Additionally, a substantial proportion of patients in the HIIT and MICT groups achieved hsCRP levels of less than 1 mg/L, indicative of a low risk of developing cardiovascular complications [76]. This underscores the clinical significance of the exercise’s anti-inflammatory effects. Numerous studies have demonstrated that exercise regimens targeting cardiovascular health, such as HIIT or MICT, primarily induce reductions in pro-inflammatory markers, including hsCRP [77, 78, 79]. Our findings suggest that HIIT may be more efficient in reducing hsCRP levels compared to MICT, consistent with the findings of some prior studies [79, 80]. Nevertheless, it’s worth noting that recent meta-analyses have not yielded conclusive evidence regarding whether HIIT consistently outperforms traditional MICT in terms of its impact on inflammatory states [78, 81]. Furthermore, limited research has explored the interplay between hsCRP levels and exercise programs specifically within the context of CAD patients.

Regarding the adherence of CAD patients to our programs, we report that only one patient in each group discontinued the intervention, reaching 96% adherence in both protocols (HIIT and MICT). Importantly, these exercise regimens demonstrated a commendable safety profile, with no reported adverse events during the exercise interventions. Our study boasts several notable strengths. It adhered to a randomized design, employed objective outcome measures, and featured blinded assessors to minimize bias. Additionally, the training interventions were thoughtfully individualized while maintaining consistent relative intensity in accordance with the HIIT principle. The favorable efficacy outcomes are particularly encouraging, given the substantial and clinically relevant improvements achieved within a relatively brief timeframe of six weeks, with a total of 18 sessions per patient. Collectively, these findings underscore the HIIT protocol as a safe, effective, and enjoyable tool for CAD patients, holding promise for enhancing their rehabilitation and overall well-being.

### Study Limitations

This study includes certain limitations that should be acknowledged. Firstly, the relatively small sample size raises the possibility that only more substantial differences would attain statistical significance. Secondly, the unintended gender bias observed in the patient cohort, with only 13–17% representation of women, poses a limitation in terms of the generalizability of the findings. It is important to note that the sex distribution in the study was an unintended consequence of our clinical population composition. When considering the results of this study, due consideration must be given to potential confounding effects stemming from concurrent medications, although it is crucial to highlight that no alterations in medication dosages for lipid-lowering and heart rate control occurred throughout the study duration. Furthermore, it is noteworthy that the control group participants were not provided with diaries, thereby rendering us devoid of information regarding their physical activity patterns during the intervention period spanning from baseline to the six-week mark. The potential increase in physical activity within the control group could introduce a mitigating factor, potentially diminishing the observed differences in effects between the various groups.

## 5. Conclusions

In summary, our randomized controlled study demonstrated that both six-week HIIT and MICT programs were not only safe but also effective in eliciting favorable outcomes concerning blood pressure, body composition, and blood biomarkers in cardiac patients. Particularly noteworthy was the HIIT group’s superior performance compared to the conventional community-based exercise program (MICT), displaying enhancements in SBP, reductions in total body fat mass, abdominal fat percentage, and waist circumference, as well as improvements in lipid profiles, blood glucose levels, and T3 hormone concentrations among patients with CAD. Conversely, the absence of any exercise-based intervention post-cardiac event correlated with adverse outcomes across all clinical variables. Importantly, no adverse events were reported, supporting the inclusion of HIIT as a valuable adjunct or alternative to MICT within community-based exercise programs, positioning it as a significant therapeutic strategy for managing CAD patients.

## Data Availability

The data that support the findings of this study are available from the corresponding author, CG, upon reasonable request.

## Supplementary Material

Supplementary material associated with this article can be found, in the online version, at https://doi.org/10.31083/j.rcm2503102.
