# Supplementary material for: Effects of High-Intensity Interval Training vs Moderate-Intensity Continuous Training on Body Composition and Blood Biomarkers in Coronary Artery Disease Patients: A Randomized Controlled Trial
Source: Rev Cardiovasc Med. 2024 Mar 11;25(3):102. doi: 10.31083/j.rcm2503102 (PMC11263861; doi:10.31083/j.rcm2503102)
Supplement: Supplementary file 1 [file 2153-8174-25-3-102-s1.docx]

**Supplementary Material**

Supplementary Table 1. Means (standard errors) of patients’ heart rate and rate of perceived exertion (Borg scale) averaged across sessions and weeks for high-intensity interval training (HIIT) and moderate-intensity continuous training (MICT).

|  | **HIIT** | | | | | | **MICT** | | | | | |
| --- | --- | --- | --- | --- | --- | --- | --- | --- | --- | --- | --- | --- |
|  | **1** | **2** | **3** | **4** | **5** | **6** | **1** | **2** | **3** | **4** | **5** | **6** |
| **HR** | 146 (3) | 138 (1) | 138 (2) | 136 (2) | 137 (3) | 134 (1) | 127 (3) | 124 (2) | 124 (3) | 120 (1) | 123 (2) | 119 (2) |
| **RPE** | 7 (0.4) | 6 (0.4) | 7 (0.2) | 6 (0.3) | 6 (0.3) | 5 (0.2) | 6 (0.4) | 6 (0.3) | 6 (0.3) | 6 (0.2) | 5 (0.4) | 5 (0.3) |

HR = Heart rate (beats per minute); RPE = Rating of perceived exertion (Borg scale 0–10).
